# ST-segment depression in atrioventricular nodal reentrant tachycardia: Important finding or just an artifact?

**DOI:** 10.1097/MD.0000000000031806

**Published:** 2022-12-09

**Authors:** Jakub Mercik, Jadwiga Radziejewska, Katarzyna Pach, Grzegorz Zawadzki, Dorota Zyśko, Jacek Gajek

**Affiliations:** a Department of Emergency Medicine, Wroclaw Medical University, Poland; b Klodzko County Hospital, Poland; c Students’ Scientific Association, Department of Emergency Medical Service, Wroclaw Medical University, Poland; d Department of Emergency Medical Service, Wroclaw Medical University, Poland.

**Keywords:** AVNRT, ST-segment depression, tachycardia

## Abstract

The ST segment is component of the QRS-T complex located between the QRS and the T wave. ST segment changes during tachycardia with narrow QRS mainly takes the form of ST segment depression. This phenomenon is often observed in young healthy people for whom an ischemic background is unlikely.

**Materials and methods:**

The study included 104 patients (71 women and 33 men) with paroxysmal narrow QRS complex tachycardia. In all patients electrophysiological study was performed and the diagnosis of atrioventricular nodal reentrant tachycardia was established. The arrhythmogenic substrate was then eliminated successfully by subsequent ablation using radiofrequency energy which confirmed the diagnosis, all patients had measured QRS components – QR, RS and RJ during the tachycardia and during the sinusrhythm. All of the measurements were done in lead V5.

**Results:**

The difference RJ-QR during tachycardia and sinus rhythm correlated negatively with tachycardia cycle length (*R* = 0.356, *P* = .001), first slowly, then rapidly reaching the cycle value of about 300 ms, then it decreases, stabilizing at the cycle level of about 270. By separating the RJ-QR in tachycardia and in the sinus rhythm from the tachycardia cycle, we can see that the correlation described in this point is largely due to the correlation between the heart rate and RJ-QR length in tachycardia.

**Conclusions:**

In patients with atrioventricular nodal reentrant tachycardia, there is a significant ST-segment depression during tachycardia episodes and the degree of this change is related to tachycardia cycle length. The most probable explanation of the ST-segment depression is the overlap of the QRS complex on the preceded T wave. This phenomenon is also influenced by some intrinsic properties of the individual electrocardiogram. It is possible to rule out ischemic origin of the presented ST segment change.

## 1. Introduction

The ST segment is a component of the QRS-T complex located between the QRS and the T wave. Physiologically, it forms an isoelectric line. ST segment abnormalities result from changes in position relative to the isoelectric line and the duration and direction of myocardial repolarization. If they arise during the period of repolarization, such as in ischemia or myocarditis, they are called primary changes. If, on the other hand, they result from changes in the depolarization period, such as in bundle branch block, myocardial ventricular pacing or pre-excitation, they are called secondary changes.^[1]^ Since ST-segment changes are 1 of the most important parameters in the diagnosis of ischemia, it is important to understand all the possibilities influencing its presence, which may lead to misinterpretation of the electrocardiogram (ECG).^[2]^

ST-segment changes are commonly seen in patients with narrow-complex paroxysmal tachycardia who have not had previous symptoms of ischemic heart disease. This population is dominated by young patients without a history of chronic diseases and no risk factors for ischemic heart disease to explain this abnormality on the ECG. In this group of patients, the terms tachycardia-induced ST-segment depression, myocardial subendocardial ischemia, recording artifact, and other ill-defined terms are used to explain these ECG changes.^[3–5]^ These ST segment alterations may occur in the presence of various types of artifacts that overlap with the individual components of the QRS complex, which alter the baseline and imitate the typical electrocardiographic changes seen in myocardial ischemia.^[6]^ An example of such changes is atrial flutter in the inferior leads, in which the flutter wave can imitate pathological q waves and resemble a previous myocardial infarction, but may also affect the ST segment and T waves, mimicking acute ischemic changes. In atrioventricular reentrant tachycardia (AVRT), the retrograde (usually negative) P wave may overlap not only with the ST segment, but also with the ascending T wave.^[7]^ In addition to the P wave, the presence of an accessory atrioventricular (AV) conduction pathway may also result in the presence of artifacts (e.g., QRS morphology, amplitudes).^[8]^ In AV nodal reentrant tachycardia, the retrograde P wave, due to the much shorter conduction through the AV junction, and not cell-to-cell conduction as in AVRT, is much more likely to be found on the descending component of the QRS complex, or in the S wave, if present. Therefore, it is usually not visible.^[9]^

Therefore, the most likely explanation for ST-segment depression is related to the overlapping of the individual components of the heart cycle with a sufficiently rapid tachycardia cycle. Thus, the T wave overlaps with the following QRS complex raising the baseline in front of the QRS complex, which results in the visual appearance of ST segment depression.

## 2. Aim

The aim of this study was to assess the presence and possible mechanisms of ST segment depression during atrioventricular nodal reentrant tachycardia (AVNRT) in patients undergoing radiofrequency ablation of the underlying arrhythmia.

## 3. Material and methods

The study included 104 patients (71 females and 33 males; mean age 50 years) with clinically important paroxysmal narrow QRS complex tachycardia. An electrophysiological study was performed in all the patients and the diagnosis of atrioventricular nodal reentrant tachycardia was established. The arrhythmogenic substrate was then successfully eliminated by ablation using radiofrequency energy. The clinical and demographic characteristics as well as laboratory tests of the patients are presented in Table 1.

**Table 1:** Clinical characteristics of the total population of studied patients.

	Total
	N = 104
Mean age (years)	50.06 ± 16.18
Male/female	33/71
Comorbidities:	_
HT	64 (61.5%)
DM	16 (15.4%)
CKD	0 (0%)
IHD	7 (6.7%)
HF	3 (2.9%)
Laboratory	_
Hemoglobine (mmol/L)	14.02 ± 1.31
K^ + ^(mmol/L)	4.41 ± 0.40
Glucose (mg/dL)	101.59 ± 22.4
Creatinine (mg/dL)	0.8 ± 0.13
TSH (mU/L)	1.95 ± 1.49
Mean denivelation (RJ-QR Tachycardia – RJ-QR NSR) (mV)	0.058 ± 0.065

CKD = chronic kidney disease, DM = diabetes mellitus, HF = heart failure, HT = arterial hypertension, IHD = ischemic heart disease.

During electrophysiological study, the cycle length of the sinus rhythm and tachycardia, as well as the amplitudes of the QRS components – QR, RS and RJ during the tachycardia and during the sinus rhythm with a paper speed of 200 mm/s and an enhancement of 64 to 128x were measured. Despite the presence of ST depressions in many leads, the measurements were made in lead V5 due to the consistent best visibility (highest amplitude) of the R wave and the most pronounced ST changes.

### 3.1. Statistical analysis

The statistical analysis was performed using the computer program STATISTICA v.13.3 (StatSoft, Inc, Tulsa, USA). For quantitative variables, basic descriptive statistics were calculated and the compliance of their distributions with the theoretical normal distribution was checked using the Shapiro–Wilk’s *W* test. Comparisons were performed with the Wilcoxon signed-rank test for dependent groups. The correlations between the studied parameters were performed using Spearman’s rank correlation coefficient according to statistical properties of the data. *P* values < .05 were considered significant.

Generalized additive models (GAMs) were used to assess the shape of the curve which fit best with the data. GAMs’ objective is to find the curve that minimizes the so-called “penalized sum of squares,” which is the sum of squares known from linear regression plus a “penalty” for wiggliness proportional to the integral of the squared second derivative. Minimalization is done within a very large set of curves that is formulated from the “basis”: a small set of functions that can generate almost every smooth curve through linear combinations. “Penalty” prevents GAMs from generating a perfect fit (a curve that goes through every data point). Such a formulation allows for the interpretation of a fitted curve as a best compromise between a perfect fit and concise description of an analyzed relationship. GAMs can also result with a linear fit, which proves that the linear relationship is the 1 that best summarizes the connection between the analyzed variables.

The study was approved by the local Bioethical Committee at the Wroclaw Medical University number KB – 213/2020.

## 4. Results

The basic electrocardiographic measurements in sinus rhythm and tachycardia are presented in Table 2.

**Table 2:** The basic electrocardiographic parameters measurements in sinus rhythm and tachycardia with according differences and statistical test results.

	QR (mV)	RS (mV)	RJ (mV)	RJ – QR (mV)	Cycle length (ms)
Tachycardia	0.689+/−0.293	0.938+/−0.369	0.820+/−0.364	0.131+/−0.097	369.71+/−72.2
Sinus Rhytm	0.718+/−0.300	0.947+/−0.351	0.795+/−0.362	0.077+/−0.077	739.55+/−179.5
Difference	0.029	0.009	−0.025	−0.054	–
*P*	.005	.771	.088	.001	–

The tachycardia-related changes in patients with AVNRT include the elevation of the reference point as indicated by diminished QR amplitude as well as the depression of the J point. This influenced the difference in RJ-QR resulting in the ECG ST-segment depression.

**Figure 1.**
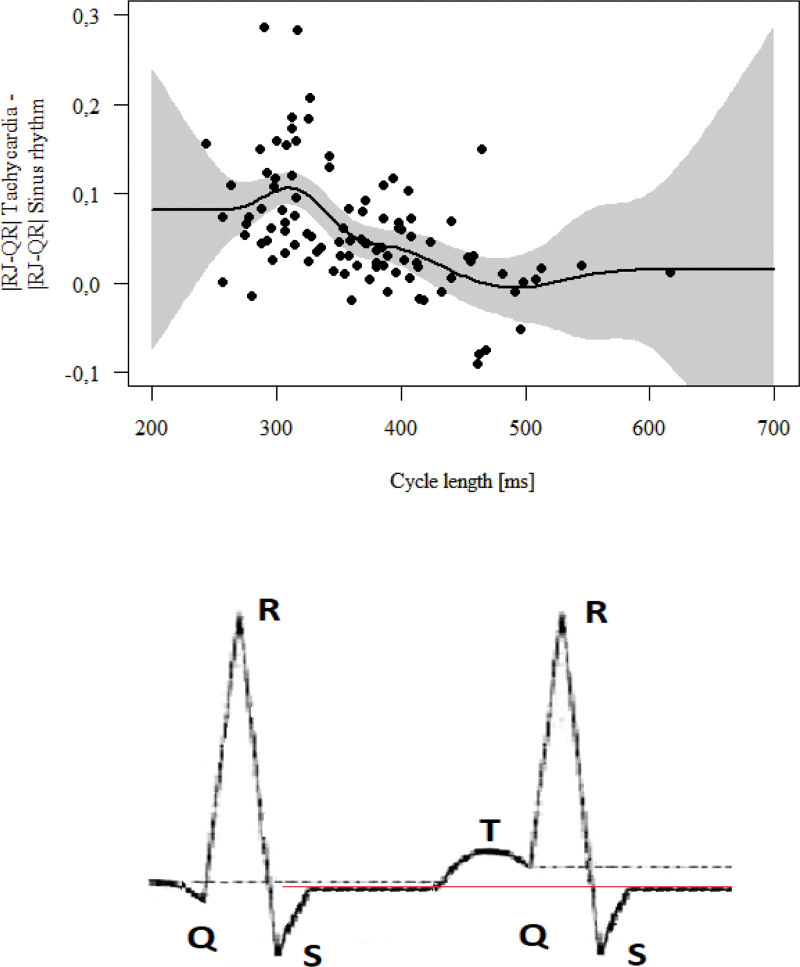
Upper panel: The RJ-QR difference with relation to the tachycardia cycle length. Lower panel: Schematic depiction of ST segment locations – dotted lines – different reference points, red line – stable ST segment level.

The difference in RJ-QR during tachycardia and sinus rhythm correlated negatively with tachycardia cycle length (*R* = 0.356, *P* = .001). The RJ-QR difference increases as the tachycardia cycle decreases, first slowly, then rapidly reaching a cycle value of about 300 ms, then it decreases, stabilizing at the cycle level of about 270. The entire graph looks like the tip of the QRS complex and the T wave, overlapping the next evolution. This relationship is shown in Figure 1.

**Figure 2.**
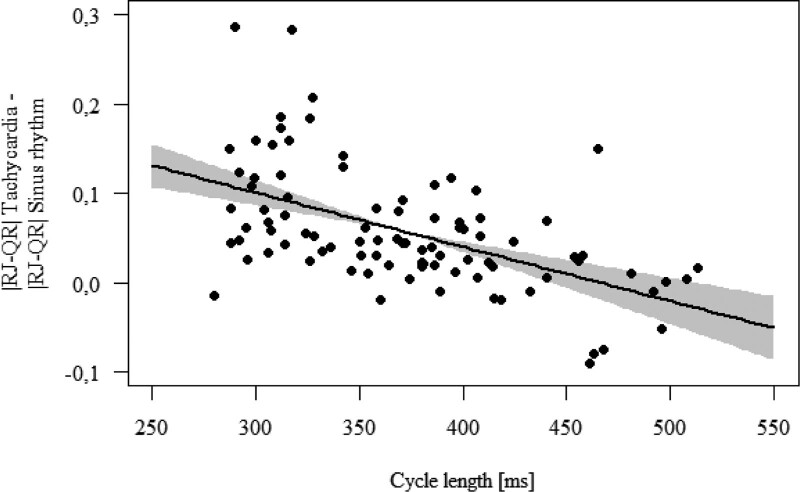
The almost linear relationship of RJ-QR difference with relation to the tachycardia cycle length when narrowing down the tachycardia cycle length.

By narrowing down the tachycardia cycle length between 280 and 520 ms, the relationship is practically linear, as shown in Figure 2 below.

By separating the RJ-QR in tachycardia and in the sinus rhythm from the tachycardia cycle, we can see that the correlation described in this point is largely due to the correlation between the heart rate and RJ-QR length in tachycardia; the correlation with the RJ-QR length in sinus rhythm is statistically insignificant. Those values are shown in Table 3.

**Table 3:** The correlation of RJ-QR in tachycardia and sinus rhythm with tachycardia cycle length.

Parameters	Heart rate in tachycardia
	Spearman correlation rank
RJ-QR Tachycardia	*r* = −0.356, *P* = .001*
RJ-QR Sinus Rhythm	*R* = 0.074, *P* = .484

**Figure 3.**
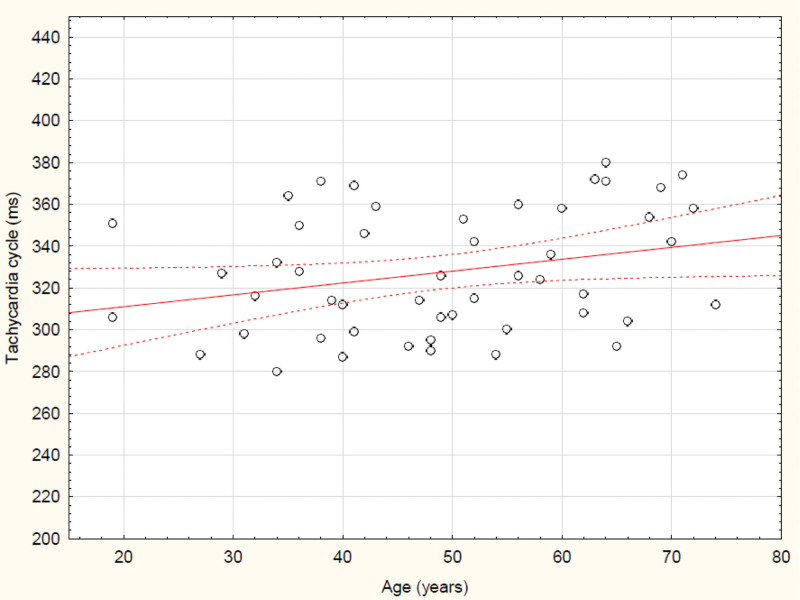
The positive correlation between the tachycardia cycle length and the age of the studied patients (*R* = 0.28, *P* = .043).

The relationship between heart rate and patient age is shown in Figure 3 below. The older the patient is, the slower the tachycardia.

## 5. Discussion

The phenomenon of ST segment depression during atrioventricular nodal reentrant tachycardia has been described in the literature, however, there is no comprehensive explanation for this finding. Many potential mechanisms of ST segment depression during tachycardia have been provided. In our opinion, the most important mechanism is the overlap of the T wave with the subsequent QRS complex. Currently, there is no definitive data for a detailed explanation of this phenomenon in the literature. Some studies suggest that heart rate is the main mechanism for the ST-segment depression.^[10]^ Our study found that the most likely mechanism is a decrease in the amplitude of the QR component with no change in the RJ component, resulting in a strong correlation between the RJ-QR difference during sinus rhythm and tachycardia.

These conclusions are further confirmed by the correlation between the depression of the ST segment and the length of the tachycardia cycle, as well as the correlation between the latter parameter and patient age. Heart rate during the tachycardia decreases with age and the ST segment depression is not age-related. Therefore, the most likely mechanism for the changes seen in ST depression in these conditions is purely electrocardiographic, and not ischemic.

Other mechanisms responsible for ST segment alterations are as follows:

### 5.1. Manifestation of myocardial ischemia during tachycardia

Several studies have shown that the occurrence of recurrent tachycardia is associated with underlying myocardial ischemia. Bukkapatnam et al studied 104 patients admitted with a diagnosis of supraventricular tachycardia (SVT), 80 of whom had troponin I testing, and 70 of these patients could be assessed for ST-segment changes.^[11]^ Thirty-seven patients (48%) had increased troponin I (mean 1.54 +/− 2.7 ng/dL, normal < 0.07 ng/dL) and 46 patients (57%) had ST-segment depression > 1.0 mm. More females than males showed a significant increase in troponin during tachycardia (55 vs 32). Among patients with a history of coronary heart disease before the onset of tachycardia, 62% had a significantly increased levels of cardiac troponins compared with 43% in patients without a history of coronary artery disease (CAD). In all patients with elevated cardiac troponin levels, chest pain was as common as in patients with troponin levels within the normal range (35% vs 32%). Among all patients, 44% had CAD verified by performing coronary angiography. Patients with CAD were older and had a higher frequency of risk factors for ischemic disease: hypertension, hyperlipidemia, and renal failure. Neither chest pain nor an increase in troponin I during SVT was significantly associated with CAD. The evidence for CAD was detected in 22% of patients displaying ST depression compared to patients without ST depression. In conclusion, there were no significant differences in baseline characteristics and clinical presentation of patients with and without troponin I increase or ST-segment depression. There was no difference in the diagnosis of CAD by noninvasive or invasive testing in patients with and without increased troponin I.^[11]^ A group of 16 patients with documented tachycardia with narrow QRS complex with significant ST segment depression (> = 1 mm) in whom a treadmill exercise test (modified Bruce protocol) was performed was studied by Petsas et al.^[12]^ ST depression below 1 mm was considered significant if it occurred up to 80 ms from the J point. A positive test result occurred only in 1 female, while in all the other patients, it was not possible to achieve ST-segment depression similar to those observed during tachycardia. These results suggest that coronary artery disease and myocardial ischemia are not involved in the genesis of ST segment depression during SVT.^[12]^ Gulec et al studied 39 patients (23 females and 16 males) admitted to the emergency department with paroxysmal SVT.^[13]^ No patient had clinical evidence of CAD, and 12-lead resting electrocardiograms were normal. Patients were divided into 2 groups according to whether they had ST-segment depression during paroxysmal SVT. Group I included 21 patients who had ST-segment depression of at least 1 mm at 80 ms after the J point in any lead during episodes of paroxysmal SVT. Group II consisted of 18 patients without ST-segment depression during paroxysmal SVT. Patients were scored according to their coronary risk factors including hypertension, diabetes mellitus, smoking, hypercholesterolemia, and a family history of ischemic heart disease. Each patient had an ECG assessed during tachycardia, during exercise testing, and during thallium stress scintigraphy; 6 patients had a positive exercise test and 7 patients had a positive thallium stress scintigraphy. In all patients, significant coronary artery disease was confirmed by coronary angiography. There was no statistically significant difference in the occurrence of chest pain, heart rate and magnitude of ST-segment depression in patients with and in patients without coronary artery disease. Only the difference in the presence of risk factors was statistically significant between patients with and without coronary artery disease.^[13]^ Nelson et al, sought to determine the relationship between supraventricular tachycardia and the symptoms of myocardial ischemia in 19 patients with inducible orthodromic atrioventricular reciprocating tachycardia.^[14]^ Sixteen of these patients had a single accessory pathway and 3 patients had dual accessory pathways. Six patients had inducible AV nodal reentrant tachycardia. A second group of 7 patients with multivessel coronary artery disease and exertional angina was included in this study to evaluate whether atrial pacing-induced ST segment depression would result in myocardial lactate production. In these patients, relatively modest ST-segment depression was associated with lactate production, indicating an ischemic substrate, in contrast to patients with supraventricular tachycardia in whom even significant ST depression was not associated with lactate production.^[14]^ The findings from these studies, suggest that the best way to determine whether tachycardia reveals significant ischemic changes is to follow the patient’s age and the presence of significant coronary artery disease risk factors.

### 5.2. ST segment changes and QT interval

Another factor influencing ST segment changes is the duration of the QT interval. During its shortening, when at a certain heart rate “it does not keep up” and overlaps the next QRS complex, it raises the baseline of the initial part of the QRS complex and results in the visual phenomenon of ST segment depression. Physiologically, the QT interval is shorter during tachycardia – the faster the tachycardia, the longer the QT interval. It is associated with adrenergic stimulation and the release of catecholamines from the myocardium. The duration of the QT interval is also gender dependent; females at any given age have a significantly longer corrected QT interval than males.^[15]^ Therefore, in females, changes in the ST segment, thought to be a measurement artifact, should appear with slower tachycardia than in males. There is also a relationship between age and QT interval duration – the older the patient, the shorter the QT interval.^[16]^ It appears that the main factor responsible for the change in the QT interval is the interval from the beginning of the QRS complex to the peak of the T wave; the rest of the QT complex is less sensitive to changes related to the heart rate.^[17]^ According to a recent study, the QT segment maintains a specific hysteresis – as the heart rate accelerates, it shortens slowly at first, then faster and faster, while when decelerating, it first shortens quickly, then slower and slower until it is extended to the initial QT.^[18]^ This phenomenon occurs because the QT interval responds with some delay to changes in heart rate. However, the exact mechanisms responsible for shifting the QT interval remain unknown and will require further investigation.

### 5.3. U wave and the overlapping of the following QRS complex

The U wave is the end of the T wave. A normal U wave should be consistent with the T wave. U waves with the opposite direction may indicate myocardial ischemia^[19]^ and should be best seen in the leads from the right ventricle. The interval between the peak of the T wave and the peak of the U wave should not exceed 150 ms.^[20]^ During tachycardia, the baseline may be raised, especially in those that are not very rapid, in which the QRS complex overlaps the U wave but not yet overlaps the T wave.

### 5.4. St-segment changes and artifacts: retrograde p-wave and „saw teeth” in atrial flutter

During atrioventricular node reentrant and atrioventricular reentrant tachycardia, a retrograde P wave projecting on the ST segment may be seen on some ECGs. It is more often visible in AVRT due to the longer RP’ interval (usually about 100 ms), while in the AVNRT and its most common form (slow-fast), it is usually less present. This is why in AVNRT its influence alter rather the shape of the QRS complex – the descending arm of the R wave and the S wave than the ST segment.^[9]^

Another example of this phenomenon is the atrial flutter wave. In a typical counterclockwise version, the flutter wave is mainly seen in the inferior leads and can overlap the ST segment and the T wave causing the baseline to rise and the reference point to change, which may resemble a depression of the ST segment. These changes are even more visible if the patient takes I C antiarrhythmic drugs, for example, propafenone, which by prolonging the arrhythmia cycle can cause 1:1 conduction.^[21]^

Another interesting aspect of ST changes involves ventricular extrasystole. There are some reports that in the QRS preceding ventricular extrasystole, mainly those originating in the right ventricular outflow tract, changes in the ST segment may be seen, mainly in the form of depression.^[22]^

In view of our results, and those from other studies in the literature, we conclude that the main mechanism responsible for the ST segment change during AVNRT is the overlapping of the previous evolution T wave with the next evolution R wave. It is actually an ECG artifact resulting from the change of the reference point, namely the rise of the baseline. This effect is only seen in those tachycardias that are fast enough for overlapping of the components to occur.

## 6. Limitations

Most of the patients participating in the study were relatively young adults, but there were also middle-aged and some elderly patients. In our study, we did not test for troponin levels during tachycardia as well as after its termination to determine ischemia, or perform coronary or angio computed tomography to assess for the presence of CAD.

## 7. Conclusions

1. In patients with AVNRT, the ST-segment is depressed during episodes of tachycardia, and the degree of this change is related to the tachycardia cycle length in particular between cycle length 280 to 520 ms.
2. The most probable explanation of the ST-segment depression is the overlapping of the QRS complex on the preceded T-wave, changing the reference point (baseline) for observation and measurement.
3. The ischemic origin of these ST-segment changes could be excluded in these patients.

## Author contributions

**Conceptualization:** Jakub Mercik, Jadwiga Radziejewska, Katarzyna Pach, Grzegorz Zawadzki, Dorota Zyśko, Jacek Gajek.

**Data curation:** Jakub Mercik, Jadwiga Radziejewska, Katarzyna Pach, Grzegorz Zawadzki, Dorota Zyśko, Jacek Gajek.

**Formal analysis:** Jakub Mercik, Jadwiga Radziejewska, Katarzyna Pach, Grzegorz Zawadzki, Dorota Zyśko, Jacek Gajek.

**Funding acquisition:** Jakub Mercik, Jadwiga Radziejewska, Katarzyna Pach, Grzegorz Zawadzki, Dorota Zyśko, Jacek Gajek.

**Investigation:** Jakub Mercik, Jadwiga Radziejewska, Katarzyna Pach, Grzegorz Zawadzki, Dorota Zyśko, Jacek Gajek.

**Methodology:** Jakub Mercik, Jadwiga Radziejewska, Katarzyna Pach, Grzegorz Zawadzki, Dorota Zyśko, Jacek Gajek.

**Project administration:** Jakub Mercik, Jadwiga Radziejewska, Katarzyna Pach, Grzegorz Zawadzki, Dorota Zyśko, Jacek Gajek.

**Resources:** Jakub Mercik, Jadwiga Radziejewska, Katarzyna Pach, Grzegorz Zawadzki, Dorota Zyśko, Jacek Gajek.

**Software:** Jakub Mercik, Jadwiga Radziejewska, Katarzyna Pach, Grzegorz Zawadzki, Dorota Zyśko, Jacek Gajek.

**Supervision:** Jakub Mercik, Jadwiga Radziejewska, Katarzyna Pach, Grzegorz Zawadzki, Dorota Zyśko, Jacek Gajek.

**Validation:** Jakub Mercik, Jadwiga Radziejewska, Katarzyna Pach, Grzegorz Zawadzki, Dorota Zyśko, Jacek Gajek.

**Visualization:** Jakub Mercik, Jadwiga Radziejewska, Katarzyna Pach, Grzegorz Zawadzki, Dorota Zyśko, Jacek Gajek.

**Writing – original draft:** Jakub Mercik, Jadwiga Radziejewska, Katarzyna Pach, Grzegorz Zawadzki, Dorota Zyśko, Jacek Gajek.

**Writing – review & editing:** Jakub Mercik, Jadwiga Radziejewska, Katarzyna Pach, Grzegorz Zawadzki, Dorota Zyśko, Jacek Gajek.

## References

[1] Deshpande A Birnbaum Y. ST-segment elevation: Distinguishing ST elevation myocardial infarction from ST elevation secondary to nonischemic etiologies. World J Cardiol. 2014;6:1067–79. 25349651 10.4330/wjc.v6.i10.1067 PMC4209433

[2] Nimmermark MO Wang JJ Maynard C. The impact of numeric and graphic displays of ST-segment deviation levels on cardiologists’ decisions of reperfusion therapy for patients with acute coronary occlusion. J Electrocardiol. 2011;44:502–8. 21871996 10.1016/j.jelectrocard.2011.06.009

[3] Dorenkamp M Zabel M Sticherling C. Role of coronary angiography before radiofrequency ablation in patients presenting with paroxysmal supraventricular tachycardia. J Cardiovasc Pharmacol Ther. 2007;12:137–44. 17562784 10.1177/1074248407300775

[4] Jastrzȩbski M. ST-segment depression and elevation during supraventricular tachycardias. Kardiol Pol. 2012;70:291–93. 22430416

[5] Kozlowski D Kozluk E Adamowicz M. Histological examination of the topography of the atrioventricular nodal artery within the triangle of Koch. Pacing Clin Electrophysiol. 1998;2:163–7. 10.1111/j.1540-8159.1998.tb01081.x 9474665

[6] Takayanagi K Hoshi H Shimizu M. Pronounced ST-segment depression during paroxysmal supraventricular tachycardia. Jpn Heart J. 1993;34:269–78. 8411633 10.1536/ihj.34.269

[7] Tai CT Chen SA Chiang CE. A new electrocardiographic algorithm using retrograde P waves for differentiating atrioventricular node reentrant tachycardia from atrioventricular reciprocating tachycardia mediated by concealed accessory pathway. J Am Coll Cardiol. 1997;29:394–402. 9014995 10.1016/s0735-1097(96)00490-1

[8] Arya A Kottkamp H Piorkowski C. Differentiating atrioventricular nodal reentrant tachycardia from tachycardia via concealed accessory pathway. Am J Cardiol. 2005;95:875–8. 15781021 10.1016/j.amjcard.2004.12.020

[9] Rivera S De La Paz Ricapito M Conde D. The retrograde P-wave theory: explaining ST segment depression in supraventricular tachycardia by retrograde AV node conduction. Pacing Clin Electrophysiol. 2014;37:1100–5. 24697871 10.1111/pace.12394

[10] Kim Y Sousa J el-Atassi R. Magnitude of ST segment depression during paroxysmal supraventricular tachycardia. Am Heart J. 1991;122:1486–7. 1951022 10.1016/0002-8703(91)90601-d

[11] Bukkapatnam RN Robinson M Turnipseed S. Relationship of myocardial ischemia and injury to coronary artery disease in patients with supraventricular tachycardia. Am J Cardiol. 2010;106:374–7. 20643248 10.1016/j.amjcard.2010.03.035

[12] Petsas AA Anastassiades LC Antonopoulos AG. Exercise testing for assessment of the significance of ST segment depression observed during episodes of paroxysmal supraventricular tachycardia. Eur Heart J. 1990;11:974–9. 2282927 10.1093/oxfordjournals.eurheartj.a059637

[13] Güleç S Ertaş F Karaoŏuz R. Value of ST-segment depression during paroxysmal supraventricular tachycardia in the diagnosis of coronary artery disease. Am J Cardiol. 1999;83:458–60, A10. 10072244 10.1016/s0002-9149(98)00888-1

[14] Nelson SD Kou WH Annesley T. Significance of ST segment depression during paroxysmal supraventricular tachycardia. J Am Coll Cardiol. 1988;12:383–7. 3392331 10.1016/0735-1097(88)90410-x

[15] Burke JH Ehlert FA Kruse JT. Gender- specific differences in the QT interval and the effect of autonomic tone and menstrual cycle in healthy adults. Am J Cardiol. 1997;79:178–81. 9193019 10.1016/s0002-9149(96)00707-2

[16] Pearl W. Effects of gender, age, and heart rate on QT intervals in children. Pediatr Cardiol. 1996;17:135–6. 8662028 10.1007/BF02505201

[17] Kannankeril PJ Harris PA Norris KJ. Rate-independent QT shortening during exercise in healthy subjects: terminal repolarization does not shorten with exercise. J Cardiovasc Electrophysiol. 2008;19:1284–8. 18665873 10.1111/j.1540-8167.2008.01266.x PMC2811883

[18] Gravel H Jacquemet V Dahdah N. Clinical applications of QT/RR hysteresis assessment: A systematic review. Ann Noninvasive Electrocardiol. 2018;23:e12514. 29083088 10.1111/anec.12514 PMC6931600

[19] Nagayoshi Y Yufu T Yumoto S. Inverted U-wave and myocardial ischemia. QJM. 2018;111:493. 29432574 10.1093/qjmed/hcy025

[20] Surawicz B Childers R Deal BJ. AHA/ACCF/HRS recommendations for the standardization and interpretation of the electrocardiogram: part III: intraventricular conduction disturbances: a scientific statement from the American Heart Association Electrocardiography and Arrhythmias Committee, Council on Clinical Cardiology; the American College of Cardiology Foundation; and the Heart Rhythm Society: endorsed by the International Society for Computerized Electrocardiology. . Circulation. 2009;119:e235–40. 19228822 10.1161/CIRCULATIONAHA.108.191095

[21] Pastromas S Sakellariou D Koulouris S. Atrial flutter with 1:1 atrioventricular conduction and profound nonischemic ST segment depression. Hosp Chronicles. 2011;6:131–33.

[22] Kukla P Sławuta A Jastrzębski M. Unusual changes in ventricular repolarization before right ventricular outflow tract arrhythmias. Am J Med Sci. 2017;353:311–2. 28262222 10.1016/j.amjms.2016.11.009

